# Techno-economic assessment for the production of algal fuels and value-added products: opportunities for high-protein microalgae conversion

**DOI:** 10.1186/s13068-021-02098-3

**Published:** 2022-01-18

**Authors:** Matthew Wiatrowski, Bruno C. Klein, Ryan W. Davis, Carlos Quiroz-Arita, Eric C. D. Tan, Ryan W. Hunt, Ryan E. Davis

**Affiliations:** 1 Catalytic Carbon Transformation and Scale-up Center, National Renewable Energy Laboratory, 15013 Denver West Parkway, Golden, CO 80401 USA; 2 Biomass Science and Conversion Technologies, Sandia National Laboratories, Livermore, CA 94550 USA; 3 Algix, 5168 Water Tower Rd, Meridian, MS 39301 USA

**Keywords:** Techno-economic assessment, Microalgae, High-protein, Conversion, Biofuels, Bioproducts

## Abstract

**Background:**

Microalgae possess numerous advantages for use as a feedstock in producing renewable fuels and products, with techno-economic analysis (TEA) frequently used to highlight the economic potential and technical challenges of utilizing this biomass in a biorefinery context. However, many historical TEA studies have focused on the conversion of biomass with elevated levels of carbohydrates and lipids and lower levels of protein, incurring substantial burdens on the ability to achieve high cultivation productivity rates relative to nutrient-replete, high-protein biomass. Given a strong dependence of algal biomass production costs on cultivation productivity, further TEA assessment is needed to understand the economic potential for utilizing potentially lower-cost but lower-quality, high-protein microalgae for biorefinery conversion.

**Results:**

In this work, we conduct rigorous TEA modeling to assess the economic viability of two conceptual technology pathways for processing proteinaceous algae into a suite of fuels and products. One approach, termed mild oxidative treatment and upgrading (MOTU), makes use of a series of thermo-catalytic operations to upgrade solubilized proteins and carbohydrates to hydrocarbon fuels, while another alternative focuses on the biological conversion of those substrates to oxygenated fuels in the form of mixed alcohols (MA). Both pathways rely on the production of polyurethanes from unsaturated fatty acids and valorization of unconverted solids for use as a material for synthesizing bioplastics. The assessment found similar, albeit slightly higher fuel yields and lower costs for the MA pathway, translating to a residual solids selling price of $899/ton for MA versus $1033/ton for MOTU as would be required to support a $2.50/gallon gasoline equivalent (GGE) fuel selling price. A variation of the MA pathway including subsequent upgrading of the mixed alcohols to hydrocarbon fuels (MAU) reflected a required solids selling price of $975/ton.

**Conclusion:**

The slight advantages observed for the MA pathway are partially attributed to a boundary that stops at oxygenated fuels versus fungible drop-in hydrocarbon fuels through a more complex MOTU configuration, with more comparable results obtained for the MAU scenario. In either case, it was shown that an integrated algal biorefinery can be economical through optimal strategies to utilize and valorize *all* fractions of the biomass.

**Supplementary Information:**

The online version contains supplementary material available at 10.1186/s13068-021-02098-3.

## Background

The establishment of a global-scale biobased economy will create a need for novel feedstocks and processes aiming at the supply of fuels and products. From this standpoint, microalgae production and conversion in integrated units may play a significant role in this network. Adding value to all microalgae biomass fractions in a biorefining setup is imperative for achieving viability in the current market. Although commercializing microalgae-derived biofuels at a competitive price of $2.50/GGE as per the 2030 target set by the US Department of Energy (DOE) would represent a technological breakthrough, relying on fuel alone as the primary source of revenue for the biorefinery is not likely feasible in view of the technical challenges and high biomass costs involved [1, 2]. The possibility of maximizing biomass value through the extraction of very high added value products is often limited by species, cultivation strategy, and other factors [3], as well as by their market size, which hinders the application of this strategy in a scenario of multiple commodity fuel-scale plants. Therefore, coupling fuel production with niche products, e.g., pigments, omega-3 fatty acids, and specialty polysaccharides [4] (while such products may enable a nascent algae industry in the near term), presents risks of saturating a small market and would not be a long-term sustainable concept to support commodity production volumes. The premise, however, changes for commodity chemicals and other compounds with otherwise substantial market sizes given a more elastic demand.

Historical attempts at adding value to microalgae biomass in this way targeted lipids and carbohydrates as the preferred starting algal constituents for conversion since they readily yield products of industrial interest with a minimum amount of (industrially established) operations, such as esterification/hydrodeoxygenation (HDO) for lipids and fermentation for carbohydrates, with numerous options for subsequent upgrading to fuels or higher-value chemicals [1]. The unsaturated fatty acid portion of the lipid fraction has also gained recent interest as a precursor for alternative products such as polyurethane, a widely used polymer with a broad range of possible applications [5, 6]. Such developments were based on microalgae with “premium” compositional profiles, i.e., low-protein, high-carbohydrate, moderate-lipid content obtained with less nutrient-replete cultivation/harvesting conditions. More recently, increased attention has been given to high-protein microalgae. Despite a less appealing compositional profile for industrial use (i.e., lower levels of carbohydrates and lipids), high-protein algal biomass benefits from lower cultivation residence times to harvest and accordingly higher productivities and (usually) lower production costs [7]. In addition to the fact that nutrient-replete growth of microalgae will more readily facilitate achieving future productivity and biomass cost goals, it justifies new attempts at adding value to it through sequential deconstruction and conversion of biomass.

Exploring new coproduct opportunities through innovative approaches is also a way of lowering the burden on the farm side of the biorefinery, alleviating the pressure to deliver microalgae biomass with high quality and at acceptable prices. In conventional biorefining approaches, protein fractions of biomass are usually routed to applications with simpler downstream processing, such as anaerobic digestion or animal feed/human food applications [2, 8–14]. These are among the simplest possible outlets for this compound class, and in some cases may be warranted given low processing costs, large market sizes, and/or attractive selling prices.

Another approach that may be suited for high-protein biomass conversion that has been more recently investigated leverages a pathway termed mild oxidative treatment and upgrading (MOTU). The strategy aims to convert proteins and carbohydrates in a single step, with an ensuing array of operations dedicated to upgrading the obtained reaction intermediate products, carboxylic acids, into hydrocarbon fuel. While the MOTU technology has been assessed previously, focusing on high-carbohydrate microalgae biomass, it is flexible enough to process high-protein microalgae as well [6, 15]. Alternatively, high-protein microalgae biomass can undergo a biochemical processing pathway, which may share a number of commonalities with the MOTU pathway, to yield a different product portfolio. Model organisms such as *Escherichia coli* may employ selected amino acids as intermediates in metabolic pathways linked to the synthesis of alcohols and acids [16]. In this fermentative route, both soluble carbohydrates and proteins can be preferentially converted to a slate of mixed alcohols (MA), also known as fusel alcohols [17–19]. Fusel alcohols are a potential alternative biofuel because of several desired properties in comparison to ethanol, such as higher energy density and lower volatility [20]. This route is an inventive way of adding value to a biomass fraction usually destined for anaerobic digestion or animal feed while increasing liquid fuel yields from high-protein biomass. Some prior techno-economic assessment (TEA) studies have initially addressed the use of this technology for specific feedstocks [21, 22].

**Fig. 1:**
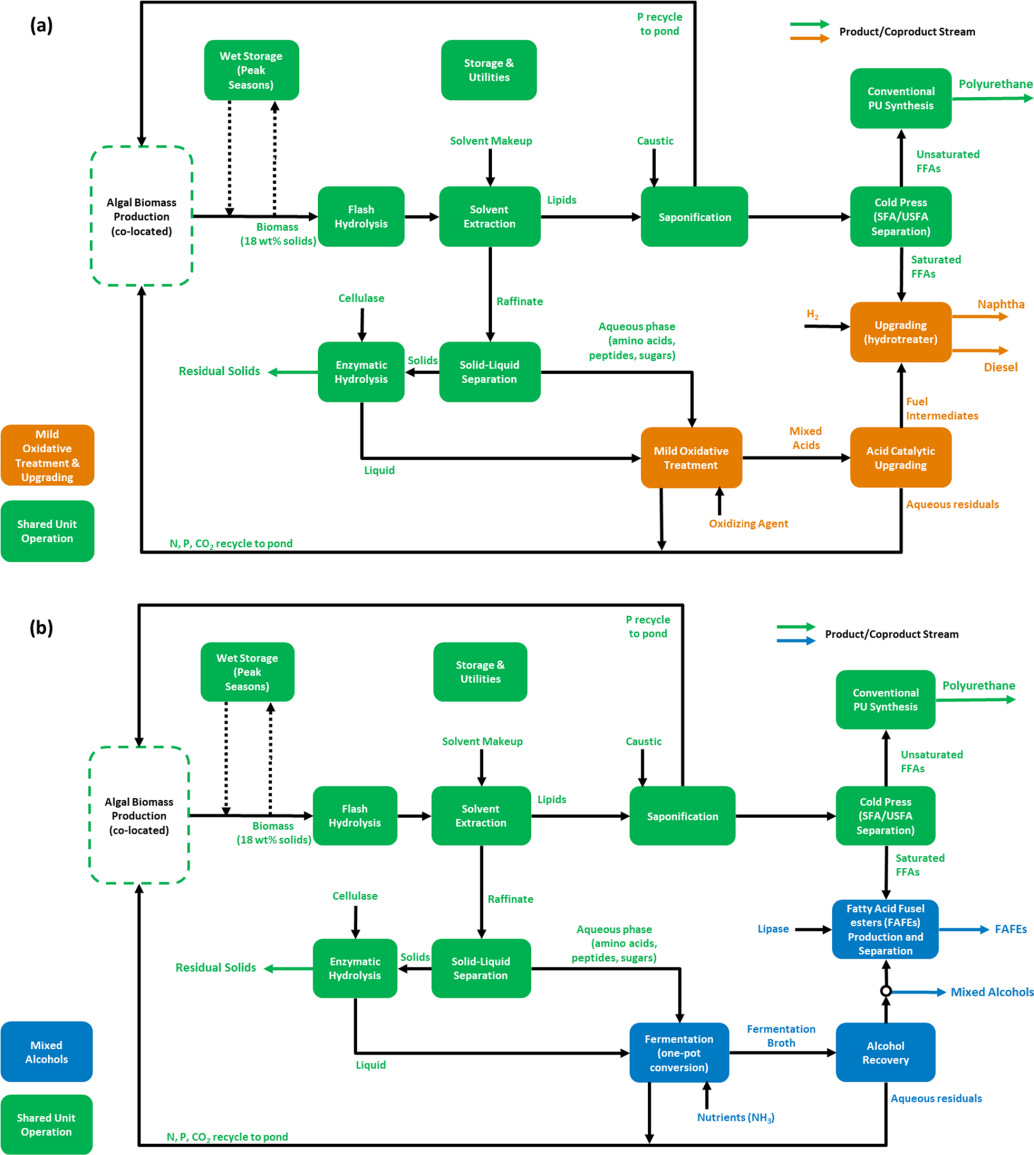
Process flow diagrams of biorefineries assessed in this study: **a** MOTU pathway and **b** MA pathway. Green blocks represent unit operations that are shared between pathways. Orange and blue boxes refer to sections specific to MOTU and MA, respectively. Dashed lines represent a fluctuating diversion of flow based on seasonal variability

In light of key potential benefits discussed above for cultivation logistics and biomass costs attributed to nutrient replete cultivation and harvesting, this effort aims at providing an updated, quantitative TEA of two conceptual routes for processing high-protein microalgae biomass in industrial facilities: MOTU- and MA-based biorefineries for fuels and bioproducts (Fig. 1). An alternative scenario including additional catalysis steps for mixed alcohol upgrading (MAU) to hydrocarbon fuels is also considered, though is relegated to a sensitivity case given more uncertainties around the associated catalytic upgrading details. In addition to fuels, focus will be given on harnessing the potential of protein-rich residual solids to be commercialized as precursors to algae-based plastics following a route currently being pursued industrially, while lipid-derived polyurethane foam is also sold as an additional coproduct.

## Results and discussion

A summary of the results for each pathway is shown in Table 1; more detailed information regarding the capital expenditures for each process can be found in Additional file 1: Table S3. Given the similarities between the two pathways examined, several commonalities are observed in each respective analysis. First, given relatively low fuel yields, both pathways rely heavily on the revenue generated from the high-value coproducts (polyurethane and residual solids), which provide the majority of the biorefinery revenues. Despite significant differences in coproduct yields within each case (with the solid coproduct yields exceeding PU by a factor of 4 for either pathway), revenue from coproducts is roughly evenly split between both polyurethane and the residual solids. This can be attributed to a large difference in coproduct value; the value of polyurethane foam ($2.04/lb) is equivalent to over $4000/ton, on the order of 4–5× greater than the calculated value of the solid coproduct. Nutrient recycle credits made up another 9–10% of revenue from the conversion biorefinery standpoint, although in the context of a fully integrated system with upstream cultivation, this is more appropriately viewed as a nutrient cost savings for the algal biomass production stage.

**Table 1:** Key techno-economic metrics of the assessed biorefining pathways

	MOTU	MA
Minimum solid coproduct selling price ($/dry ton) to support $2.5/GGE fuel price	$1033	$899
Fuel yield (GGE/AFDW ton)	34.9	44.6
Fuel yield (MMGGE/yr)	6.6 (0.3 naphtha, 6.3 diesel)	8.4 (6.9 alcohols, 1.5 FAFE)
Fuel C/O molar ratio	n/a (negligible oxygen content)	5.1 (4.6 alcohols, 11.3 FAFE)
Solid coproduct yield (lb/AFDW ton)	1009	1009
Polyurethane coproduct yield (lb/AFDW ton)	254 (140)^a^	254 (140)^a^
Carbon utilization (% of algal carbon)		
Fuel	19.6%	25.2%
Naphtha	0.9%	–
Diesel	18.7%	–
Mixed alcohols fuel	–	20.6%
FAFE	–	4.6%
Solid coproduct	38.2%	38.2%
Polyurethane (algal carbon only)	11.0%	11.0%
Total	68.9%	74.4%
Revenue breakdown (% of total)		
Fuel	7%	10% (8% alcohols, 2% FAFE)
Solid coproduct	42%	38%
Polyurethane	41%	43%
Nutrient recycle	10%	9%
Fixed capital investment ($MM)	$290 MM	$251 MM
Raw materials, utilities, and waste ($MM/yr)	$178 MM/yr	$174 MM/yr
Feedstock	60.0%	61.5%
Enzymes^b^	1.6%	2.0%
Natural gas	5.9%	7.7%
Electricity	4.3%	4.3%
Polyurethane inputs	15.8%	16.2%
Catalysts	2.0%	n/a
H_2_	0.7%	n/a
Other chemicals	9.7%	8.2%

^a^First number includes total mass of polyurethane, including diisocyanate co-reactant and other chemicals; number in parenthesis includes only mass from algae feedstock

^b^Cellulase for MOTU pathway; cellulase and lipase for MA pathway

A dependence on coproducts has been commonly observed in prior analyses on algal biorefineries when focused on producing fuels at economical cost goals [1, 6, 23]. Still, this is even more pronounced when considering high-protein biomass given lower fuel yields at least compared to standards otherwise possible for lower-protein/less-replete algae (on the order of 80–100 GGE/ton or more [23]. Fuel yields and required residual solids coproduct selling prices were relatively comparable between the two pathways, with the MA pathway producing 44.6 GGE/ton total fuel products versus 34.9 GGE/ton for the MOTU pathway, requiring a solids coproduct sales price of $899/ton for MA versus $1033/ton for MOTU (AFDW basis) in order for either approach to support a $2.5/GGE fuel price.

This increased need for coproduct revenue comes from a number of factors unique to high-protein biomass. When considering the modeled feedstock composition of the high-protein biomass, the components that are assumed convertible in either pathway comprise 81% of the biomass (carbohydrates, proteins, and lipids on an AFDW basis, as shown in Table 2). The same convertible fraction of the high-carbohydrate biomass considered in prior analyses makes up 95% of the AFDW biomass, thus adding a higher fraction of “inert” material to the biomass, which is costed but not convertible to fuels [6]. Additionally, the feedstock cost of the high-protein biomass is approximately 18% higher than the high-carbohydrate biomass considered previously due to the increased cost of nutrients for more nutrient-replete, higher-N/P content biomass at the time of harvest. Beyond the practicality advantage of nutrient-replete high-protein biomass being associated with higher achievable productivities, from the standpoint of the conversion facility, this means that a higher price must be paid *(for a given cultivation productivity)* for a feedstock that is ultimately less convertible and generally less flexible to fuel/product opportunities than those afforded by upgrading of carbohydrates and lipids in high quantities. This issue is partially addressed in these processes by realizing value for some of those non-convertible fractions as relegated to a portion of the solid residual coproduct. Additionally, much of the incremental increase in biomass production costs attributed to higher nitrogen/phosphorous nutrient demands are offset by accordingly increased recycle of those nutrients from the conversion processes back to cultivation (64% and 90% of N and P nutrients respectively are recycled from the MOTU pathway, and 53% and 90% from the MA pathway).

**Table 2:** Modeled algal feedstock composition, based on a weighted average of multiple strains across all months of cultivation reflecting year-long outdoor test-bed cultivation campaigns conducted under the DISCOVR consortium [34]

Elemental, ash free dry weight (AFDW)	Average composition (wt %)
C	51.5
H	7.6
O	30.2
N	9.3
S	0.2
P	1.2
Total	100.0
Component (dry wt%)	
Ash	11.0
Protein	40.0
Lipids^a^	9.2
Non-fuel polar lipid impurities	5.5
Fermentable carbohydrates	19.3
Other carbohydrates	3.6
Cell mass	11.4
Total	100.0

^a^Reported as FAME, roughly equivalent to the portion of lipids convertible to fuels

**Fig. 2:**
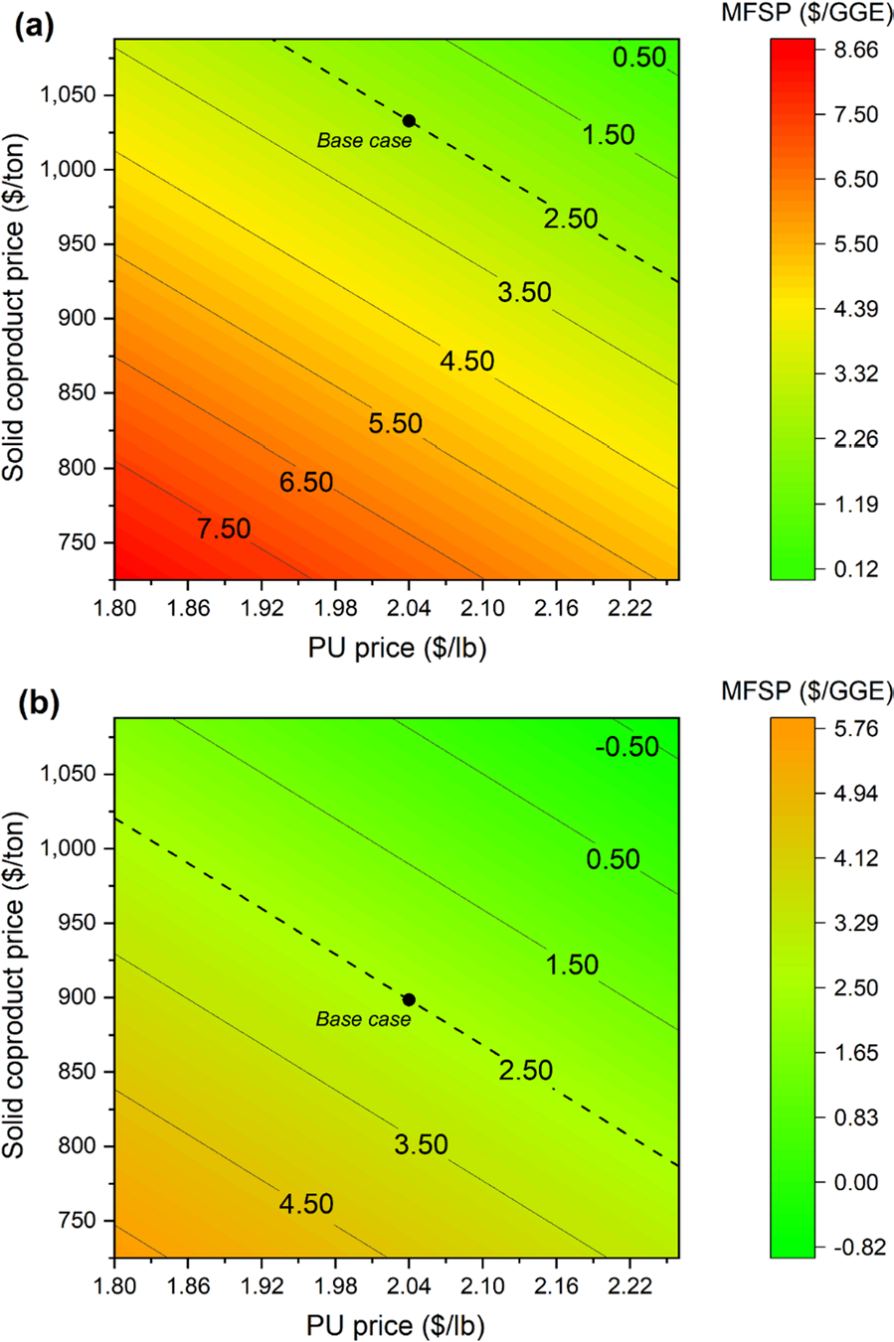
Sensitivity analysis of MFSP vs. solid coproduct and PU prices for **a** MOTU and **b** MA pathways

Having demonstrated a significant reliance of process economics on coproduct revenue, it follows that the economic sensitivity to the modeled coproduct values is of great interest. In the base case, the selling price of flexible polyurethane foam is fixed at $2.04/lb, and a solid coproduct price required to support fuel selling prices of $2.5/GGE is determined ($1033/ton and $899/ton for MOTU and MA, respectively). To analyze the sensitivity of the process to the coproduct values, a range of selling prices for each coproduct was also considered. The PU price was varied over a range of $1.80/lb (representing the minimum price from the last 5 years) to $2.26/lb (a value on the upper range of prices that may be found in published literature) [1, 24]. Similarly, the solid coproduct selling price was varied over a range of $725–$1088/ton, representing a range of potential values in the context of bioplastics production based on industry guidance. The impact of these coproduct values on MFSP is shown in Fig. 2 for each pathway, further highlighting the impact this factor carries on overall economics. Higher coproduct prices enable lower fuel prices for both pathways, with significant penalties observed when either coproduct value is reduced from base values. This relationship is more pronounced for the MOTU pathway due to lower fuel yields relative to the MA pathway.

However, it is important to highlight that the MA pathway yields two types of oxygenated fuel components (fusel alcohols and FAFEs), with a combined C/O ratio of 5.1. This is an important distinction from the MOTU-based configuration [6, 25], in which the fuel products are hydrocarbons that undergo additional processing to deoxygenate fuel intermediates (lipids and MOT products) through substantial catalytic upgrading and HDO operations. The absence of catalysts in the MA pathway is responsible for a reduction of $3.6 MM/year in catalyst costs alone, besides $1.2 MM/year in hydrogen. In total, expenses for the MOTU pathway exceed those of the MA pathway by $4 MM/year for operating costs, while the associated capital costs are $39MM higher. Additionally, the MOTU pathway incurs considerable carbon losses as light gases and CO_2_ throughout the downstream catalytic upgrading steps, leading in part to the lower final fuel yields. The MA pathway’s comparatively lower MFSPs, as shown in Fig. 2, must be qualified by the fact that the fuel products are not directly comparable, and those of the MA pathway are not drop-in fuels compatible with today’s infrastructure, thus may be more limited in their market inclusion if constrained for use as fuel blending additives. Additional processing steps may similarly be added to upgrade the mixed alcohol fuels to hydrocarbons, which is considered as a sensitivity study later, but is not reported as part of the main base case results here as the details for fusel alcohol catalytic upgrading are not as well-established.

Regardless of the fuel upgrading pathway, the economic viability of either process is contingent on the ability to valorize residual biomass solids. Given this dependence, we justify rationalizing the required solids coproduct values based on guidance from industry. As an example of a pertinent commercial use of this residual material, Algix, LLC has developed thermoplastic co-processing to incorporate protein-rich, finely milled algae biomass powder into a variety of thermoplastic compounds for use in consumer products. The process is best suited to convert algae that may have limitations for conversion into biofuels, foods, feeds, or nutraceuticals due to composition or contamination (e.g., algae used for wastewater treatment or harvested from wild algae blooms) [26]. Algix has been focused on building a branded product line of thermoplastic compounds for use in a range of consumer products such as footwear, sports, and lifestyle products as well as automotive applications. These compounds contain approximately half algae biomass by weight and can displace up to 30% of the plastic content in the finished molded or foamed material. Algix also produces 100% bio-based and compostable compounds by blending algae biomass with compostable resins which have the potential to be used in packaging, agriculture, and 3D printing.

For the creation of thermoplastic compounds, the algal biomass can be valorized at a price of $800–$1000 per metric tonne ($725–$910/ton) and potentially extending up to $1200 per metric tonne ($1088/ton) for solids of sufficient quality. The algae biomass specification for thermoplastic compounding requires at least 30% protein, less than 35% ash/mineral content, less than 20% carbohydrates, less than 10% lipids, and less than 10% moisture. These specifications are met in the TEA modeling designs evaluated in this study, at 31% protein, 24% ash, 19% carbohydrates, < 1% lipids, and 10% or less moisture (following cellulase hydrolysis to selectively reduce carbohydrates). The market for algae infused products is rapidly increasing as consumer brands and companies continue to adapt and increase their sustainability initiatives for reducing the environmental and societal impacts of their products and services. Thus, direct utilization of the algae biomass through thermoplastic compounding provides an early entry-point into the market, opening the door for a range of other bio-products, as algal biomass production becomes more ubiquitous around the world.

An additional sensitivity considers the final disposition of residual solids. Both pathways utilize an enzymatic treatment step with cellulase to meet Algix quality specifications on protein and carbohydrates when focused on utilizing the residual solids for this purpose. This step selectively solubilizes additional carbohydrates present in the unconverted solids and diverts them to MOT/MA fermentation for further conversion to fuel, resulting in a higher fuel yield at the expense of (more valuable) solid coproduct. As a sensitivity, a scenario was also analyzed where the separated solids were dried directly without any additional treatment. The solids in this scenario, containing 26% protein and 32% carbohydrates by weight, do not meet specifications for the Algix process but may be alternatively valorized as animal or fish feed components. When evaluated through the MOTU pathway, the minimum solid coproduct price for this scenario reduces considerably to $792/ton, as compared to a price of $1033/ton for the base case, while for the MA pathway the required selling price drops to $778/ton in comparison to $899/ton for the base case. This impact can generally be attributed to a 20% increase in solid coproduct mass yield, which delivers a higher value than fuel at these levels, as well as savings associated with the removal of enzymatic hydrolysis. There is also a notably higher overall carbon utilization efficiency for maintaining and maximizing solids to this coproduct stream rather than diverting a portion to fuel: whereas fuel production requires more complex and costly processing operations while losing carbon along the way, the solids can simply be dried and sold. This not only achieves 100% carbon retention from the unconverted solids but also allows for additional mass contributions from components that would not otherwise contribute to fuel. Of course, this relies on the solid coproduct meeting market quality demands, which is not a guarantee given the presence of ash, salts, etc., but is beyond consideration in the current scope of analysis (no further processing steps are included in the TEA calculations for this sensitivity case). Higher-value animal feeds may be sold comparably to soy protein on the order of $500/ton, with fish feeds up to $1000/ton or more [27].

Considering the relative merits and challenges for the technologies considered here, the MOTU pathway benefits from a non-oxygenated hydrocarbon fuel product, at the expense of lower fuel yields and increased processing costs. The lower fuel yields are a result of losses incurred through the upgrading train used to upgrade the oxygenated carboxylic acids to hydrocarbon fuels. The most significant loss of carbon is observed in MOT, where 53% of carbon present in the protein and carbohydrates is converted to ketonizable acids, with the remainder effectively lost as CO_2_ (some is converted through MOT to formic acid, which is then lost to CO_2_ in ketonization). Additional losses in the form of CO_2_ are observed in ketonization, where each reaction of two carboxyl groups results in the release of one molecule of CO_2_. Finally, moderate amounts of carbon are converted to offgas in the hydrotreating of SFAs. While these cracking reactions occur in the same reactor as the upgrading of the condensation products, the latter are only mildly oxygenated and are assumed to be upgraded to paraffins without any further loss of carbon.

In contrast, many of the merits of the MA pathway are related to the simpler carbon conversion processes and leaner downstream operations. The ability of *E. coli* to uptake amino acids as an additional carbon source effectively more than doubles the output of alcohols in comparison to a case in which carbohydrates are the only available carbon source. Even at low alcohol titers (kept at under 20 g/L to avoid toxicity effects on the fermenting microorganism), the recovery of mixed alcohols is achieved with several atmospheric distillation columns and a relatively low energy consumption. The enzymatic synthesis of FAFEs also benefits from a straightforward process that achieves high lipid conversions in small reactors operating at low temperatures and recovers esters with operations commonly found in biodiesel production plants. The FAFE product also achieves higher mass yields by way of oxygen retention, compared to the hydrocarbon product from lipid hydrotreating in MOTU; however, similar to the caveats on the mixed alcohol stream, this also means the FAFE product may not be directly utilized at large scale in currently deployed infrastructure, but rather may be limited to blending restrictions similar to other biodiesel products.

Additionally, during fermentation, *E. coli* is able to use certain amino acids as convertible substrates, thus enriching the broth towards other unconverted amino acids. The pathway could also enable the valorization of those remaining unused amino acids (valine, proline, alanine, glycine, methionine, cystine, histidine, and hydroxyproline) [28]. Commercialization of part of this fraction as a higher value-added product could be achieved at the expense of a complex downstream process—often chromatographic techniques. The exploitation of purified amino acids has a clear market limitation for simultaneous deployment in multiple n^th^ plants. Still, it is an option to leverage the initial development of first-of-a-kind facilities. Alternatively, glycine could be specifically redirected to butanol and isobutanol fermentation with *Saccharomyces cerevisiae* [29], thus increasing fuel yields in a potential setup employing a consortium of microorganisms.

### Sensitivity analysis: upgrading of fusel alcohols to hydrocarbons

As a final sensitivity analysis, the TEA implications for adding a catalytic upgrading train to convert the mixed alcohol product from the MA biorefinery into hydrocarbons were also investigated (MAU case). This is of high relevance to allow for a more equitable comparison of the two biorefining approaches by ultimately constraining them to have fuel outputs with similar characteristics. Since alcohols are highly reactive moieties [30], this prospect appears as a feasible possibility, although the conversion of this specific fusel alcohol mix through multiple sequential catalytic steps still needs experimental validation to support TEA estimates, and thus is viewed as carrying somewhat more uncertainty than the upgrading details for the MOTU pathway.

**Fig. 3:**
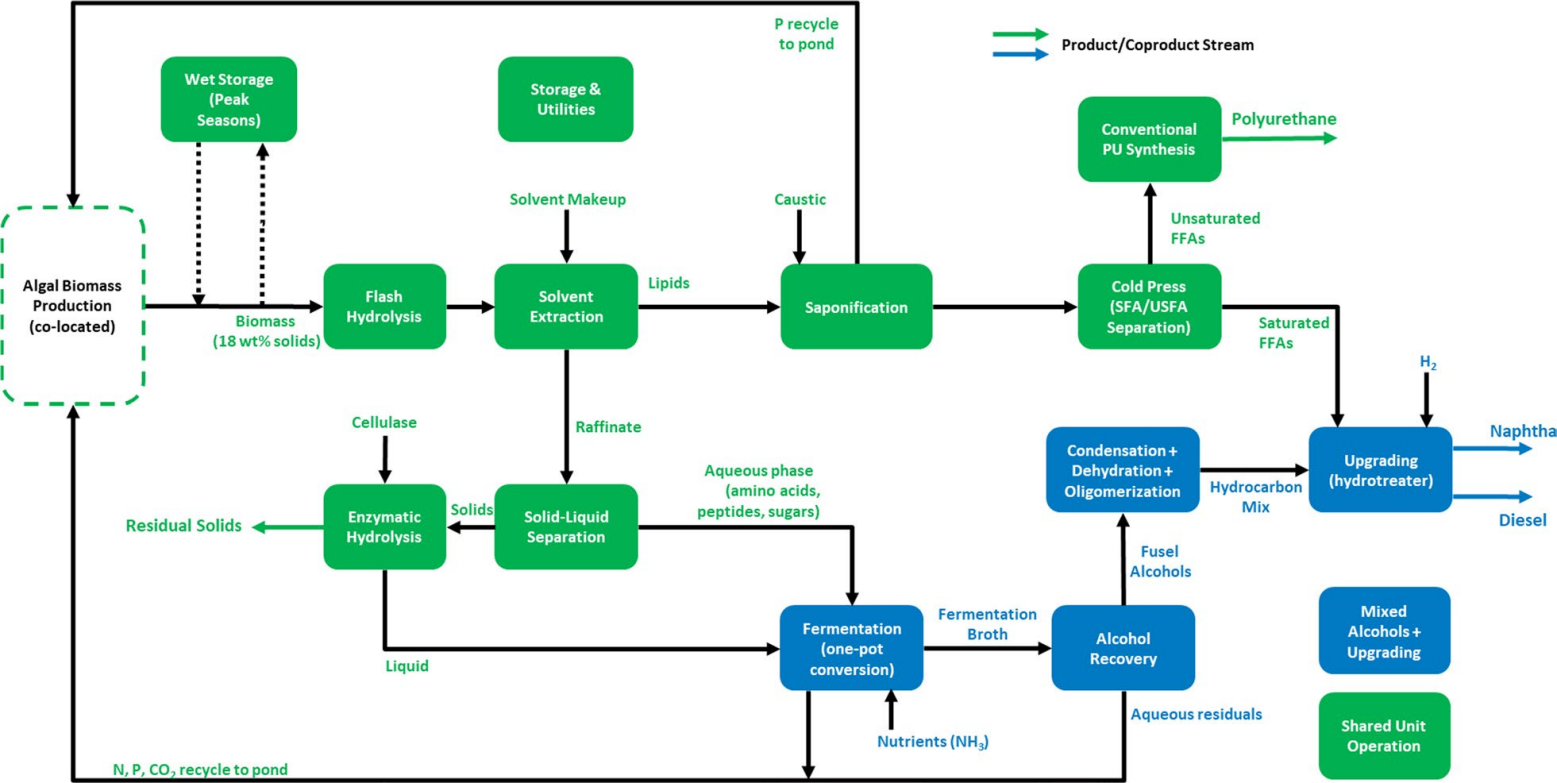
Process flow diagram of a biorefinery based on the MA pathway. Green blocks represent unit operations that are shared between this pathway and the two main biorefining configurations investigated in this study, while blue boxes refer to sections specific to the new MAU plant. Dashed lines represent a fluctuating diversion of flow based on seasonal variability

A detailed description of the catalytic upgrading process is provided in the Supplementary Material, and the main process parameters are shown in Additional file 1: Table S4. This case study maintains a similar framework for the overall MA biorefinery configuration, as shown in Fig. 3**.** In an overview, the alcohols produced in the fermentation step are recovered from the broth and fractionated into light (ethanol and isobutanol) and heavy alcohols (2-methyl-1-butanol, 3-methyl-1-butanol, and phenylethanol) so that both fractions are processed separately. Light alcohols undergo a Guerbet condensation reaction followed by dehydration; heavy alcohols are sent directly to dehydration; the dehydration products are then combined and sent to a single oligomerization reactor; finally, the hydrocarbon mix (primarily in the C12-16 range) is hydrotreated with saturated FFAs from the cold press and fractionated into naphtha and diesel fuels.

The minimum solid coproduct selling price in this MAU biorefinery scenario increases by around 8%, up to $975/ton in comparison to the simpler MA pathway ($899/ton), while fuel output is down by 4% to 42.8 GGE/dry ton (AFDW basis). In a simple analysis, there is a higher dependence on solid coproduct selling price when processing carbohydrates and proteins into a final hydrocarbon fuel. When comparing the estimated number of $975/ton for this pathway with the MOTU pathway, in which a minimum coproduct selling price of $1033/ton was found for a configuration with relatively high fuel output (34.9 GGE/AFDW ton), it can be concluded that both biorefining configurations are fairly equivalent when focused on producing hydrocarbons as the final fuel product. The reduction in operational expenses with lipase used previously for FAFE production is offset by higher costs with H_2_ for hydrotreating and catalysts. The upgrading train of mixed alcohols involves a series of sequential catalytical reactions, each one of them requiring a different catalyst. Despite processing a very small flowrate of mixed alcohols (3.3 t/h entering the alcohol upgrading section), the expenses with catalysts amount to nearly $1 MM/year. These costs are dominated by a zeolite-based platinum catalyst used for hydrotreating (81%), followed by a HZSM-23 zeolite used for oligomerization (17%). The combined cost for the four remaining catalysts amounts to 2% of the total catalyst expenses.

## Conclusions

Comprehensive TEA modeling was performed on two potential process approaches for producing fuels and coproducts from high-protein algal biomass through an integrated biorefinery schematic focused on deconstructing and converting individual biomass constituents. The two pathways share a number of commonalities, with the main difference being that the MOTU pathway follows a more complex thermochemical upgrading strategy to produce hydrocarbon fuels while the MA pathway involves a more simplistic biological approach with less processing, albeit producing oxygenated fuels. The MA pathway demonstrated moderate economic benefits compared to the MOTU pathway, highlighting potential advantages if such oxygenated molecules can be effectively implemented into the fuel market at the same price point as that of hydrocarbon fuels, and/or blended as at low levels into the hydrocarbon fuel pool. Alternatively, the MOTU pathway presents a route to achieving drop-in deoxygenated fuel blendstocks. When including similar catalytic upgrading steps to convert mixed alcohols to hydrocarbons, the MAU sensitivity case exhibited more comparable fuel yields and economics compared to the MOTU pathway.

In either case, analysis to achieve $2.5/GGE fuel selling price targets revealed a strong dependence on maximizing the utilization of all fractions of the biomass feedstock, particularly with a need to convert a significant portion of the biomass to high-value coproducts to offset high biomass feedstock costs. This dependence on coproducts is not a new finding when producing fuels from algae but is more pronounced when considering the additional challenges of converting high-protein algal biomass. While both pathways require substantial coproduct revenues to achieve economic viability alongside fuels, namely polyurethanes at $2.04/lb and valorization of residual solids for sale at $899–$1033/ton (MA and MOTU pathways, respectively), the coproducts considered in this study well exceed market volume constraints of very high-value but small-scale niche products to support commodity-scale algal biorefineries. Finally, polyurethanes represent an existing large market opportunity with potential for renewable feedstocks, while residual solids represent an attractive substrate for synthesis into novel bioplastic products.

## Methods

Rigorous process models were developed in Aspen Plus to track the overall mass and energy balances. These process models were developed according to the process designs shown in Fig. 1. In the MOTU pathway, solubilized carbohydrates and proteins undergo mild oxidative treatment (MOT) and are converted into short-chain carboxylic acids. These acids are then deoxygenated and elongated by a series of catalytic steps to yield hydrocarbon fuels in the naphtha and diesel range. In the MA pathway, the solubilized glucans and proteins are used as substrates for fermentation to a mixed alcohols slate; similar catalytic elongation/deoxygenation steps are considered for the MAU sensitivity case. Material and energy balances generated from the Aspen Plus models are utilized to inform the TEA, which entails a discounted cash flow rate of return analysis for each biorefinery.

### Description of biorefineries

Both algal biorefineries share a number of consistent unit operations and herein will be described together. The biorefinery is assumed to be co-located with an algal biomass cultivation facility supporting biomass production and harvesting/dewatering (outside the scope of TEA focus in this work). Both pathways have identical upstream processing operations, with the major variations limited to the fuel upgrading strategy. Each pathway starts with the pretreatment of algal biomass, maintained at a constant rate despite seasonal variation using a wet anaerobic storage step for peak cultivation seasons [31]. Following pretreatment, lipids are extracted from the biomass and separated into unsaturated fatty acids (USFAs), which are upgraded to a polyurethane coproduct, and saturated fatty acids (SFAs), which are upgraded to fuel. The raffinate from extraction is separated into solids and liquids, with solids undergoing further conversion before being dried for sale as a coproduct.

Solubilized protein and carbohydrates present in the liquid are then upgraded to fuels, with the upgrading strategy varying between pathways. The MOTU pathway utilizes mild oxidative treatment to produce carboxylic acids, which are catalytically upgraded to fuels, while the MA pathway produces fusel alcohols via fermentation. Fusel alcohols are subsequently recovered and either used directly as fuels or combined with SFAs to produce fatty acid fusel esters (FAFEs); the alternative FAU case includes catalytic upgrading of the fusel alcohols to hydrocarbons. Both pathways include a nutrient recycle of recovered N, P, and CO_2_ to the algae cultivation facility and account for all storage and utility needs. Many unit operations are identical to those used in prior analyses, and the details are maintained here as documented in Davis et al. [6]. Detailed technical parameters are presented in Additional file 1: Table S1.

#### Pretreatment

In both the MOTU and MA pathways, high-protein algal biomass with the composition shown in Table 2 is fed as a slurry to flash hydrolysis, following upstream dewatering to 18 wt% ash-free dry weight (AFDW) and seasonal anaerobic storage as required to normalize seasonal flows constantly throughout the year. In flash hydrolysis, the material is subjected to elevated temperatures and pressures (280 °C, 1200 psig) for a short residence time of approximately 10 s. These conditions have been shown to convert significant amounts of protein, as well as moderate amounts of carbohydrate, into water-soluble constituents [32, 33]. Proteins are converted to both polypeptides, which show more resistance to hydrolysis, as well as free amino acids. Similarly, carbohydrates are converted to monomers (i.e., glucose and mannose) and soluble oligomers. Polar lipids, consisting of polar heads and fatty acid tails, are also assumed to be partially saponified [33]. Additional details on the flash hydrolysis operation can be found in Davis et al. [6].

#### Solvent extraction

The pretreated biomass from both pathways proceeds to the lipid extraction section, where it is subjected to multiple agitation and phase separation steps, using ethanol and hexane as co-solvents. Each solvent is recovered and recycled via separate distillation units, recovering hexane from the lipid extract phase and ethanol from the aqueous raffinate phase. The remaining aqueous product, consisting of solubilized proteins and carbohydrates, proceeds to the fuel upgrading train (unique for each pathway), while the extracted lipids proceed to saponification.

#### Saponification

In previous work, algal lipids have been assumed to undergo a bleaching/degumming operation to remove impurities [6, 25]. That work focused mostly on high-carbohydrate biomass, where the lipid fraction contains a significantly lower amount of polar components. In contrast, the majority of lipids present in high-protein biomass are present as polar lipids, consisting of a polar head and a non-polar tail. This larger fraction of polar impurities necessitates the use of a saponification operation, which involves the use of water and caustic to cleave the polar heads from the lipids. A large fraction of these bonds (80%) is assumed to be cleaved in flash hydrolysis, with the remainder cleaved here. The fatty acid tails then form a soap with the cation of the caustic. A strong acid is used to neutralize the fatty acids, which then proceed to cold press separation. The aqueous phase, containing any phosphorous associated with the polar heads, is recycled to the algae ponds for nutrient recovery.

#### Cold press

A hydraulic cold press separates SFAs and USFAs by exploiting the difference in the melting point temperatures. This separation is done in a series of five steps over sequentially decreasing temperatures, reaching as low as 9 °C. It should be noted that this operation has not been practiced at a commercial scale for the purpose of SFA/USFA separation. Initial experimental results have been promising but do not yield a perfect separation. To represent a future target scenario, the models assume a complete separation of saturated and unsaturated fatty acids. Though there are no losses observed experimentally or in the model (all fatty acids are utilized for PU or fuel), SFA impurities in the USFA-rich phase could have an impact on the final properties of the polyurethane. Alternatively, a pure USFA phase, but with significant losses into the SFA fraction, would negatively impact PU yields.

#### PU synthesis

The polyol and subsequent polyurethane synthesis processes are consistent with details published previously [6]. Briefly, free fatty acids are reacted with acetic acid and peroxide in a one-pot epoxidation and ring-opening reaction, yielding polyols. Impurities in the polyol stream are removed by a series of distillation steps and the purified polyols are polymerized to flexible polyurethane foam by combination with a diisocyanate. Cutting and handling of the polyurethane foam are done on-site, with a separate storage facility added to account for curing and storage needs.

#### Raffinate clarification

Following ethanol solvent recovery, the raffinate from lipid extraction, an aqueous slurry containing solubilized carbohydrates and proteins as well as a portion of the hydrolyzed polar lipid heads, is separated into solid and liquid phases by use of a vacuum belt filter press. Solids for each pathway undergo additional treatment via enzymatic hydrolysis with cellulase. The liquid from the filter press advances to the fuel upgrading section for each pathway.

#### Solid treatment

In both MOTU and MA pathways, the unconverted solids consist of proteins, carbohydrates, ash, and “cell mass,” a mixture of chlorophyll, nucleic acids, and other unidentified components in the compositional analysis. These solids may be suitable for sale as a coproduct for a purpose such as animal feed or use as a co-feed for the synthesis of bioplastics, as pursued by the commercial company Algix; however, in the latter case, the carbohydrate content (32%) is too high for direct use in the Algix process. Therefore, an additional enzymatic treatment step is used to selectively decrease the carbohydrate content and accordingly further enriches protein to meet Algix specifications. This enzymatic hydrolysis step requires re-dilution to 20% solids prior to reaction. Preliminary experimental work has indicated promising results utilizing cellulase enzyme for this purpose (unpublished data), although an optimum enzyme dosage is not yet established; for this assessment, an enzyme loading target of 10 mg/g carbohydrates is assumed in the TEA model, achieving 50% hydrolysis of residual carbohydrates to sugar monomers.

In either pathway, the product from enzymatic hydrolysis again undergoes solid–liquid separation, with the liquid being diverted to the fuel upgrading train. The solids are dried in a double-drum dryer, yielding a dry solid coproduct suitable for use in the Algix process (or potentially for sale suitable as high-protein animal or fish feed); the boundary of TEA modeling does not include protein processing operations beyond drying, and is configured to solve for the dried solids coproduct value as required to achieve a targeted fuel price metric.

#### Upgrading: MOTU pathway

The details of the process design for MOT and catalytic upgrading are largely consistent with those published previously [6]. One main difference is that previous modeling had assumed a whole slurry processing configuration through MOT, including insoluble solids. More recent experimental results indicate that solids are more challenging to be processed and converted through MOT at desired high solids content in the slurry, likely stemming from mass transfer limitations between the oxidant and the solids. Conversely, the liquid phase containing solubilized proteins and carbohydrates is more amenable to conversion. Accordingly, in this work, we have assumed only conversion of the soluble liquor fraction, while also reducing the target carbon efficiency to carboxylic acids from 80 to 60%. Otherwise, the details of the MOT and catalytic upgrading section of the MOTU model remain unchanged with previously documented work. Briefly, oxygen is sparged into a bubble column-type reactor and is consumed along with carbohydrates and protein, yielding mixed carboxylic acids [6]. Excess heat from the exothermic reactions is used to generate a portion of the plant’s low-pressure steam requirement, and the CO_2_ produced is recycled back to the algae ponds.

The aqueous stream from the MOT reactor is first sent to an ion-exchange column to recover the nitrogen and phosphorous present in the stream. Next, the stream is heated and routed to catalytic ketonization, employing a fixed heterogeneous catalysis reactor to upgrade acids to larger chain length ketones in the C3-7 range through coupling reactions. These ketones are recovered via a flash distillation for lighter components and a liquid–liquid separation for heavier components and then sent to the condensation reactor. Here, the ketones are reacted in a batch slurry reactor in the presence of a toluene solvent, producing C6-15 cyclic enones and water. Finally, these cyclic enones are sent to hydrodeoxygenation and are subsequently separated into diesel and naphtha fractions, with the majority of the modeled compounds in the diesel range.

#### Upgrading: MA pathway

In this pathway, all unit operations through raffinate solid/liquid separation are consistent with the MOT pathway. Liquid fractions from cellulase hydrolysate clarification and from the solid/liquid separation are combined and routed to fermentation vessels in which *Escherichia coli* anaerobically converts substrate into five different alcohols: ethanol, isobutanol, 3-methyl-1-butanol (isoamyl alcohol), 2-methyl-1-butanol (active amyl alcohol), and phenylethanol [35]. The microorganism preferentially uptakes a number of amino acids while others are untouched and thus become enriched in the fermentation product broth. *E. coli* seed production is carried out with an external glucose source.

Although full fractionation of the fusel alcohol mixture is feasible with a series of distillation columns and decanters [36], the chosen configuration for this analysis takes a more simplified approach. The fermentation broth is initially routed through a large distillation column for bulk water removal, and the stream containing the mixed alcohols (with around 50 wt% water) is sent to a decanter for phase enrichment. The water-rich phase is further treated in a rectifying column for additional alcohol recovery. The overhead stream of this column is combined with the organic phase issued from the decanter and sent to a molecular sieve for final water removal, based on published literature utilizing molecular sieve dehydration for similar higher-alcohols [21, 22, 37, 38]. The majority of this finished product is sent to storage for sale as a blended alcohol fuel product (translated into total GGE fuel yield according to this stream’s lower heating value calculated in Aspen Plus). At the same time, a fraction is routed to the production of FAFEs through the reaction between saturated fatty acids and fusel alcohols. SFAs issued from the cold press are used in this conversion, thus avoiding the need for a hydrotreater in this configuration. The conversion was based on the assumptions by Monroe et al. [39], in which an *Aspergillus oryzae* lipase catalyzes the reaction at a stoichiometric alcohol excess of 4.5 for 24 h for maximized yields. The process is suitable to low-cost lipids (waste cooking oil, grease) and enables high conversion. Purification of FAFEs and unused alcohol recovery is carried out as an adaptation of conventional biodiesel processes with decanters, washing equipment, centrifuge, a flash vessel for FAFE drying, and a distillation column for alcohol recovery. The unreacted alcohol stream is finally purified with a similar setup as presented above (rectifying column and molecular sieve) and ultimately recycled to the lipase-catalyzed enzymatic conversion. FAFEs are then sold as an additional fuel product, again with resulting fuel yields combined as total GGE based on heating values.

#### Utilities

In both cases, all required utilities are accounted for in the model, including high/low pressure steam, hot oil (for high-temperature utility heat demands in excess of high-pressure steam allowances), electric power, cooling water, chilled water, plant and instrument air, the clean-in-place (CIP) system, process water, and bulk storage.

### Approach to economics

The TEA modeling approach in this work is consistent with that described in prior works [6, 40]. The mass and energy balance outputs from the Aspen Plus model were used to determine the number and size of capital equipment items needed. As process conditions and flows change, baseline equipment costs are automatically adjusted using scaling factors. These baseline costs originally were sourced from vendor quotes when available or other means such as (primarily) Aspen Capital Cost Estimator (ACCE) [41] when necessary. The details of these equipment designs have been published in prior reports [6, 25, 42].

Once equipment costs are determined, direct and indirect overhead cost factors are applied to determine a feasibility-level estimate of total capital investment (TCI) in 2016 US dollars [6]. Variable operating expenses are calculated based on raw material and utility rates from the Aspen Plus model, while fixed costs (labor, maintenance, insurance, and local taxes) are based on prior works and adjusted based on plant scale [6, 40]. The TCI, operating expenses, and fixed costs are used in a discounted cash flow rate of return analysis. A common measurement of process economics employed in prior analyses is the minimum fuel selling price (MFSP) required to obtain a net present value (NPV) of zero for the plant. Here, we also consider an alternate method, namely, setting the fuel selling price to a targeted market value ($2.50/GGE) and solving for the minimum solid coproduct selling price required to support this. Given the wide range of possible solid coproduct values, depending on the ultimate end-use, this is a useful metric for highlighting the sensitivity of the process to this variable. High level financial assumptions used for the TEA are shown in Additional file 1: Table S2. These assumptions are based on mature nth-plant operational/economic assumptions, and consistent with prior published work.

High-protein algal biomass is delivered at the conversion plant gate at a price of $575/ton AFDW, reflective of a target cultivation productivity of 25 g/m^2^/day AFDW and elemental composition outlined in Table 2 (with associated CO_2_ and fertilizer nutrient costs attributed to the given C/N/P content), combined with dewatering to 18 wt% solids, with all upstream biomass cultivation/dewatering TEA model details consistent with previously published work [2, 43]. Enzymes (*A. oryzae* lipase in the MA pathway and cellulase in both pathways) are costed according to differences in required enzyme rate via scaling from a basis of $6.16/kg of enzyme protein based on previously published work [42] (at considerably smaller scales of enzyme usage here, the cost for enzymes increases to $12.26/kg for lipase and $8.65/kg for cellulase).

Hydrocarbon fuels produced in the MOTU pathway are drop-in fuels in the range of naphtha and diesel and could be sold as such in the market. Fuel outputs from the MA-based biorefinery include mixed alcohols and FAFEs: while the former could be blended with ethanol fuel up to a maximum of 3% [44], the latter could be considered as advanced biodiesel in combination with conventional diesel fuel. Still, it is important to acknowledge the difference in fuel end-products between the two pathways, given that more costs and processing complexity are invested in the MOTU pathway for full upgrading to hydrocarbons; similar upgrading strategies could also be possible with the MA pathway to produce hydrocarbons in place of mixed alcohols as market allowances dictate, considered as an alternative sensitivity case (MAU) at the end of this paper.

Residual solids obtained after solubilization of protein and glucans should be within specification for it to be a suitable precursor to bioplastics able to displace ethylene–vinyl acetate (EVA) elastomers. Based on guidance from the industry, the limits for sale of solids residuals to be leveraged by this technology require > 30% protein, < 20% carbohydrates (preferably < 10%), and < 35% ash.

Finally, algae-based polyurethanes are commercialized at a price of $2.04/lb (2016 dollars), consistent with a 5-year average price for commodity flexible foam [6].

## Supplementary Information

**Additional file 1.** **Table S1**. Detailed technical parameters of operations employed in mixed alcohols (MA) and mild oxidative treatment and upgrading (MOTU) biorefineries. **Table S2**. Financial assumptions used in the TEA, based on a mature *n*th plant. **Table S3**. Summary of capital expenditures for the MA and MOTU pathways. **Sensitivity analysis: upgrading of fusel alcohols to hydrocarbons**. **Figure S2**. Detailed processing of mixed fusel alcohols to hydrocarbon fuels via Guerbet condensation, dehydration, oligomerization, and upgrading. **Table S4**. Parameters related to the conversion of mixed alcohols to hydrocarbons.

## Data Availability

The datasets used and/or analyzed during the current study are available from the corresponding author on reasonable request.
