# Supplementary material for: Techno-economic assessment for the production of algal fuels and value-added products: opportunities for high-protein microalgae conversion
Source: Biotechnol Biofuels Bioprod. 2022 Jan 18;15:8. doi: 10.1186/s13068-021-02098-3 (PMC8764804; doi:10.1186/s13068-021-02098-3)
Supplement: Supplementary file 1 — Additional file 1. Table S1. Detailed technical parameters of operations employed in mixed alcohols (MA) and mild oxidative treatment and upgrading (MOTU) biorefineries. Table S2. Financial assumptions used in the TEA, based on a mature nth plant. Table S3. Summary of capital expenditures for the MA and MOTU pathways. Sensitivity analysis: upgrading of fusel alcohols to hydrocarbons. Figure S2. Detailed processing of mixed fusel alcohols to hydrocarbon fuels via Guerbet condensation, dehydration, oligomerization, and upgrading. Table S4. Parameters related to the conversion of mixed alcohols to hydrocarbons. [file 13068_2021_2098_MOESM1_ESM.docx]

**Additional Material To:**

**Techno-Economic Assessment for the Production of Algal Fuels and Value-Added Products: Opportunities for High-Protein Microalgae Conversion**

Matthew Wiatrowski ^a^, Bruno C Klein ^a^, Ryan W Davis ^b^, Carlos Quiroz-Arita ^b^, Eric C D Tan ^a^, Ryan W Hunt ^c^, Ryan E Davis ^a^

^a^ Catalytic Carbon Transformation and Scale-up Center, National Renewable Energy Laboratory, 15013 Denver West Parkway, Golden, CO 80401, USA

^b^ Biomass Science and Conversion Technologies, Sandia National Laboratories, Livermore, CA 94550, USA

^c^ Algix, 5168 Water Tower Rd, Meridian, MS 39301, USA

**Table S1**. Detailed technical parameters of operations employed in mixed alcohols (MA) and mild oxidative treatment and upgrading (MOTU) biorefineries.

|  | Value | Reference |
| --- | --- | --- |
| **Wet anaerobic storage** |  |  |
| Biomass degradation | 23% | Davis et al. (2020) (1)  Wendt et al. (2019) (2) |
| **Flash hydrolysis** |  |  |
| Temperature | 280°C | Bessette et al. (2018) (3)  Consistent with Davis et al. (2020) (4) |
| Pressure | 1200 psig | Bessette et al. (2018) (3)  Consistent with Davis et al. (2020) (4) |
| Residence Time | 10 seconds | Garcia-Moscoso et al. (2013) (4)  Talbot et al. (2016) (5)  Consistent with Davis et al. (2020) (4) |
| Carbohydrate solubilization | 64% | Garcia-Moscoso et al. (2013) (4)  Talbot et al. (2016) (5)  Consistent with Davis et al. (2020) (4) |
| Protein solubilization | 10% | Garcia-Moscoso et al. (2013) (4)  Talbot et al. (2016) (5)  Consistent with Davis et al. (2020) (4) |
| Phospholipid hydrolysis | 80% | Garcia-Moscoso et al. (2013) (4)  Consistent with Davis et al. (2020) (4) |
| **Protease treatment** |  |  |
| Protease loading | 1 g enzyme/L | DeRose et al. (2019) (6) |
| Enzymatic treatment time | 16 h (+ 10 h for CIP cycle) | DeRose et al. (2019) (6) |
| **Lipid extraction** |  |  |
| Configuration | 3-stage CSTR & centrifugation with two solvents | Davis et al. (2014) (7)  Davis et al. (2020) (4) |
| Solvent loading (nonpolar: EtOH: dry biomass, by weight) | 2.7: 1.1: 1 | Davis et al. (2014) (7)  Davis et al. (2020) (4) |
| CSTR extraction residence time | 15 min | Davis et al. (2014) (7)  Davis et al. (2020) (4) |
| Temperature | 55°C | Davis et al. (2014) (7)  Davis et al. (2020) (4) |
| Lipid yield per stage | 74% - 65% - 56% | Davis et al. (2014) (7)  Davis et al. (2020) (4) |
| Overall lipid yield | 96% | Davis et al. (2014) (7)  Davis et al. (2020) (4) |
| **Solid-liquid separations** |  |  |
| Configuration | Vacuum belt filter press | Davis et al. (2018) (8) |
| Wash ratio | 5:1 by mass | Davis et al. (2018) (8) |
| Exit solids content | 36% | Davis et al. (2018) (8) |
| **Mild oxidative treatment** |  |  |
| Residence time | 60 min | Davis et al. (2020) (4) |
| Solids loading | 147 g/L | Davis et al. (2020) (4) |
| Temperature | 225°C | Davis et al. (2020) (4) |
| Pressure | 35 bar | Davis et al. (2020) (4) |
| Carbon yield |  | Davis et al. (2020) (4) |
| Formic acid | 7% | Davis et al. (2020) (4) |
| Other ketonizable acids | 53% | Davis et al. (2020) (4) |
| CO_2_ | 40% | Davis et al. (2020) (4) |
| **Ketonization** |  |  |
| Configuration | Fixed bed | Davis et al. (2018) (8)  Davis et al. (2020) (4) |
| Catalyst | 0.1% Pt on Nb_2_O_5_ | Davis et al. (2018) (8)  Davis et al. (2020) (4) |
| Temperature | 350°C | Davis et al. (2018) (8)  Davis et al. (2020) (4) |
| Pressure | 57 bar | Davis et al. (2018) (8)  Davis et al. (2020) (4) |
| WHSV | 1.7 hr^-1^ | Davis et al. (2018) (8)  Davis et al. (2020) (4) |
| Conversion | 100% | Davis et al. (2018) (8)  Davis et al. (2020) (4) |
| **Condensation** |  |  |
| Configuration | CSTR (slurry) | Davis et al. (2018) (8)  Davis et al. (2020) (4) |
| Catalyst | Nb_2_O_5_ | Davis et al. (2018) (8)  Davis et al. (2020) (4) |
| Temperature | 220°C | Davis et al. (2018) (8)  Davis et al. (2020) (4) |
| Pressure | 11.2 bar | Davis et al. (2018) (8)  Davis et al. (2020) (4) |
| Residence time | 2.5 hours | Davis et al. (2018) (8)  Davis et al. (2020) (4) |
| WHSV | 2 hr^-1^ | Davis et al. (2018) (8)  Davis et al. (2020) (4) |
| Per-pass conversion | 60% | Davis et al. (2018) (8)  Davis et al. (2020) (4) |
| **Hydrotreating** |  |  |
| Configuration | Fixed bed reactor | Davis et al. (2020) (4) |
| Catalyst | 1% Pt/SAPO-11 | Davis et al. (2020) (4) |
| WHSV | 1 hr^-1^ | Davis et al. (2020) (4) |
| **Mixed alcohols fermentation** |  |  |
| Protein uptake | 85% | SNL internal data |
| Alcohols yield from proteins | 0.450 g/g | SNL internal data |
| Alcohols breakdown from proteins (wt%) | 2-methyl-1-butanol 52%  3-methyl-1-butanol 40%  Isobutanol 8% | Estimated from Davis (2019) (9) |
| Carbohydrates conversion | 90% | SNL internal data |
| Alcohols yield from carbohydrates | 0.450 g/g | SNL internal data |
| Alcohols breakdown from carbohydrates (wt%) | Isobutanol 57%  2-methyl-1-butanol 6%  3-methyl-1-butanol 12%  Ethanol 10%  Phenylethanol 15% | Estimated from Davis (2019) (9) |
| Fermentation vessel size | 3,875 million L (1 million gal) | Davis et al. (2018) (8) |
| Fermentation time | 54 h (+ 10 h for CIP cycle) | DeRose et al. (2019) (6) |
| **FAFE production** |  |  |
| Lipase loading | 2 g enzyme/100 g TAG | Monroe et al. (2020) (10) |
| TAG conversion | 99% | Monroe et al. (2020) (10) |
| Reaction time | 24 h (+ 10 h for CIP cycle) | Monroe et al. (2020) (10) |
| **Cellulase Treatment** |  |  |
| Enzyme loading | 10 mg/g carbohydrate | NREL internal data |
| Carbohydrate conversion | 50% | NREL internal data |
| Enzymatic treatment time | 30 min | NREL internal data |
| Temperature | 140°C | NREL internal data |

CIP: clean-in-place

NREL: National Renewable Energy Laboratory

SNL: Sandia National Laboratory

**Table S2**. Financial assumptions used in the TEA, based on a mature *n*^th^ plant (4,11)

| **Financial Assumptions** | **Value** |
| --- | --- |
| Plant life | 30 years |
| Plant capacity | 187,520 tons of microalgae AFDW/year |
| Cost year dollar | 2016$ |
| Capacity Factor | 90% |
| Discount rate | 10% |
| General plant depreciation | MACR |
| General plant recovery period | 7 years |
| Steam plant depreciation | MACR |
| Steam plant recovery period | 20 years |
| Federal tax rate | 21% |
| Financing | 40% equity |
| Loan terms | 10-year loan at 8% APR |
| Construction period | 3 years |
| *First 12 months’ expenditures* | 8% |
| *Next 12 months’ expenditures* | 60% |
| *Last 12 months’ expenditures* | 32% |
| Working capital | 5% of fixed capital investment |
| Start-up time | 6 months |
| *Revenues during start-up* | 50% |
| *Variable costs during start-up* | 75% |

AFDW: ash-free dry weight

APR: annual percentage rate

MACR: modified accelerated cost recovery

**Table S3.** Summary of capital expenditures for the MA and MOTU pathways.

|  | Total Cost ($MM) | |
| --- | --- | --- |
|  | MOTU | MA |
| **Total Installed Costs** | **144.8** | **123.7** |
| *Seasonal Storage and Pretreatment* | *7.2* | *7.2* |
| *Lipid Extraction, Purification, and Separation* | *20.3* | *20.3* |
| *Hydrolysate Upgrading* | *39.0* | *22.0* |
| *Polyol/Polyurethane Production* | *51.5* | *51.5* |
| *Solid Treatment* | *15.5* | *15.5* |
| *Boiler, Utilities, and Storage* | *11.2* | *7.1* |
| **Other Direct Costs** | **36.5** | **33.5** |
| **Indirect Costs** | **108.7** | **94.3** |
| **Fixed Capital Investment** | **290.0** | **251.4** |

**Sensitivity analysis: upgrading of fusel alcohols to hydrocarbons**

The anaerobic fermentation of soluble carbohydrates and proteins can be carried out with *E. coli* to generate a slate of mixed alcohols. While an innovative way to process high-protein microalgal biomass, the commercialization of oxygenated fuels, especially of a product mix as diverse as that found in this case, is restricted by blend limits – something that could be reduced or completely bypassed if the final fuel products are comprised of renewable hydrocarbons. This variation of the mixed alcohols (MA) biorefinery makes a number of modifications as well as to consider TEA implications for adding a catalytic upgrading train to convert the mixed alcohol product into hydrocarbons (MAU pathway in the main text).

The Guerbet condensation is a possible catalytic route to accomplish the synthesis of higher alcohols in systems where lighter alcohols (e.g., C1- C4) are present (12,13). Since the production of mixed alcohols through the fermentation of carbohydrates and amino acids with *E. coli* also generates ethanol and butanol as some of the main products (14), this reaction can be seen as an important starting point of a more complex system aiming at adding value to such alcohols. Further downstream, the dehydration of alcohols, their oligomerization, and hydrotreating into fuel-range hydrocarbons has already been the object of multiple studies in the literature linked to the production of sustainable aviation fuel and other liquid fuels from renewable sources (15–18), although such efforts have focused on the conversion of “conventional” alcohols (specifically ethanol, n-butanol, isobutanol, and 2,3-butanediol) instead of complex mixtures involving fusel alcohols (2-methyl-1-butanol, 3-methyl-1-butanol, and phenylethanol).

The overall biorefining configuration of high-protein algal biomass is based on the MA biorefinery (described in the main body of the manuscript) up to the purification of mixed alcohols with a molecular sieve. The process described below refers specifically to the back-end addition for catalysis steps to convert the alcohols into hydrocarbon fuels (as detailed in Figure S2), which was modeled according to the broad concept laid out by Tan et al. (19). In this way, the production of fusel alcohol fatty esters (FAFE) has been removed from the process so the fuel mix in this updated biorefinery is completely comprised of hydrocarbons. All data pertaining to conversion/yield/selectivity, pressure, temperature, and catalyst employed in each reaction step is presented in Table S3.


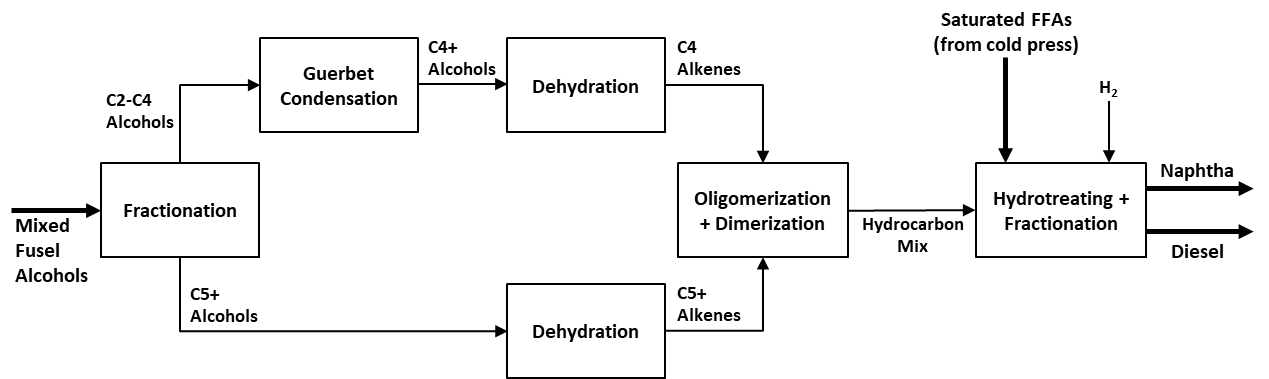
**Figure S2.** Detailed processing of mixed fusel alcohols to hydrocarbon fuels via Guerbet condensation, dehydration, oligomerization, and upgrading. Flows of off-gas, wastewater, and other residual streams are not shown for simplification purposes.

The fusel alcohol mix leaving the molecular sieves (water content of 0.5 wt%) is initially fractionated into light (ethanol and isobutanol - or C2-C4) and heavy alcohols (2-methyl-1-butanol, 3-methyl-1-butanol, and phenylethanol - or C5+) so that the Guerbet condensation of ethanol can be carried out without the interference of higher alcohols. In this initial assessment, this reaction is considered to be indispensable to minimize the presence of ethylene in the alkene mix further downstream – a compound which is significantly more difficult to oligomerize than C4 alkenes (19). Ethanol is primarily converted into C4 alcohols and, to a minor extent, to octanol and ethylene; isobutanol is also converted to C8 alcohols and isobutene; additionally, both alcohols undergo methathesis to yield branched C6 alcohols. After this, C4+ alcohols are recovered in a distillation column while the unconverted ethanol fraction and C2-C4 alkenes are removed from the loop and sent to the boiler to help partially off-set the consumption of natural gas. While the Guerbet condensation of fusel alcohols into higher alcohols could be of interest in this process to generate larger monomers for oligomerization, the main papers in the literature tend to be more focused on mixtures with high amounts of C1-C3 alcohols (12,13,20). Also, this work is not considering the (speculative) route of simply dehydrating the full alcohol mixture in a single stream, without the initial fractionation. Combining all unit operations (i.e., Guerbet condensation, dehydration, oligomerization, and hydrotreating) in a single processing train would very likely improve the economics of the biorefinery, although this option should be supported by solid experimental evidence to be eventually implemented.

In the next step, both alcohol streams (C4+ and C5+) are dehydrated separately to the respective alkenes since they require different reaction conditions (19,21). In either system, conversion is generally high (> 97%) for most alcohols. The dehydration of both amyl alcohols and phenylethanol is assumed to approach 100% over the considered bentonite catalyst (21,22). After water and residual alcohols are separated from both monomer streams, the alkene streams are combined and sent to a single oligomerization reactor. This work considers that oligomerization of C4 alkenes with isoamylene could be carried out jointly. After oligomerization, the lighter oligomers (essentially C8 alkenes) are sent to an additional dimerization reaction to enhance the yield towards C16 hydrocarbons. Finally, the heavily unsaturated hydrocarbon mix is combined with saturated FFAs from the cold press separation to undergo hydrogenation in a hydrotreating step. The resulting hydrocarbon mix is then fractionated into light and heavy cuts, which are assumed to be sold as naphtha and diesel fuels, respectively.

**Table S4.** Parameters related to the conversion of mixed alcohols to hydrocarbons.

| **Parameter** | **Values** | **References** |
| --- | --- | --- |
| **Guerbet condensation** |  |  |
| P, T | 2.4 bar, 300 ^o^C | Tan et al. (2016) (19) |
| Reactional performance | Single-pass conversion: 60% (ethanol)  Selectivities: 69.2% (butanol), 30.8% (other higher alcohols) | Tan et al. (2016) (19) |
| Catalyst type | MgO-Al_2_O_3_ | Tan et al. (2016) (19) |
| Reaction WHSV | 1 h^-1^ | Tan et al. (2016) (19) |
| Catalyst price | $26.25/lb | Tan et al. (2016) (19), catalyst cost corrected to 2016 |
| Cost recovered in reclamation | 80% | Consideration |
| Replacement period | 4 years | Tan et al. (2016) (19) |
|  |  |  |
| **C4+ dehydration** |  |  |
| P, T | 2.4 bar, 380 ^o^C | Tan et al. (2016) (19) |
| Reactional performance | Single-pass conversion: 99% (ethanol, propanol, pentanol, hexanol, heptanol), 97% (isobutanol, n-butanol), <30% (branched higher alcohols) | Tan et al. (2016) (19) |
| Catalyst type | γ-Alumina | Tan et al. (2016) (19) |
| Reaction WHSV | 1 h^-1^ | Tan et al. (2016) (19) |
| Catalyst price | $10.82/lb | Tan et al. (2016) (19), catalyst cost corrected to 2016 |
| Cost recovered in reclamation | 80% | Consideration |
| Replacement period | 3 years | Tan et al. (2016) (19) |

**Table S4 (continued)**

| **C5+ dehydration** |  |  |
| --- | --- | --- |
| P, T | 2.4 bar, 325 ^o^C | Perez et al. (1988) (21) |
| Reactional performance | 100% conversion of C5 alcohols  100% conversion of phenylethanol ^a^ | Perez et al. (1988) (21)  Hasan et al. (2012) (22) |
| Catalyst type | Bentonite | Perez et al. (1988) (21) |
| Reaction WHSV | 1.45 h^-1^ | Perez et al. (1988) (21) |
| Catalyst price | $0.14/lb | Price assumed as three times the price of conventional bentonite |
| Cost recovered in reclamation | 0% | Consideration (simple catalyst with no noble metals to be recovered) |
| Replacement period | 3 years | Consideration |
|  |  |  |
| **Oligomerization** |  |  |
| P, T | 30 bar, 250 ^o^C | Tan et al. (2016) (19) |
| Reactional performance | Single-pass conversion: 95% (butenes)  Selectivities: C8 (26.2%), C12 (43.0%), C16 (21.9%), C20+ (8.9%) | Tan et al. (2016) (19) |
| Catalyst type | HZSM-23 | Tan et al. (2016) (19) |
| Reaction WHSV | 0.21 h^-1^ | Tan et al. (2016) (19) |
| Catalyst price | $32.34/lb | Tan et al. (2016) (19), catalyst cost corrected to 2016 |
| Cost recovered in reclamation | 80% | Consideration |
| Replacement period | 3 years | Tan et al. (2016) (19) |

**Table S4 (continued)**

| **Dimerization** |  |  |
| --- | --- | --- |
| P, T | 3.7 bar, 115 ^o^C | Tan et al. (2016) (19) |
| Reactional performance | Single-pass conversion: 90% (C8 alkenes to C16 alkenes) | Tan et al. (2016) (19) |
| Catalyst type | Nafion | Tan et al. (2016) (19) |
| Reaction WHSV | 1 h^-1^ | Tan et al. (2016) (19) |
| Catalyst price | $10.38/lb | Tan et al. (2016) (19), catalyst cost corrected to 2016 |
| Cost recovered in reclamation | 80% | Assumed |
| Replacement period | 4 years | Tan et al. (2016) (19) |
|  |  |  |
| **Hydrotreating** |  |  |
| P, T | 30.6 bar, 375 ^o^C | Davis et al. (2020) (4) |
| Reactional performance | Yield to fuel-range hydrocarbons: 87.3%  *Diesel-range: 78.2%*  *Naphtha-range: 9.1%* | Davis et al. (2020) (4) |
| Catalyst type | 1% Pt/SAPO-11 | Davis et al. (2020) (4) |
| Reaction WHSV | 1 h^-1^ | Davis et al. (2020) (4) |
| Catalyst price | $360.45/lb | Davis et al. (2020) (4) |
| Cost recovered in reclamation | 42% | Davis et al. (2020) (4) |
| Replacement period | 2 years | Davis et al. (2020) (4) |

^a^ Phenylethanol dehydration to styrene is assumed to be complete. This can be carried out with a carbon-based acid catalyst (Hasan et al., 2012) (22)

**References (Supplementary Material)**

1. Davis R, Wiatrowski M. Algal Biomass Conversion to Fuels via Combined Algae Processing (CAP): 2019 State of Technology and Future Research [Internet]. National Renewable Energy Lab. (NREL), Golden, CO (United States); 2020 04-23. Available from: https://www.osti.gov/biblio/1659895

2. Wendt LM, Kinchin C, Wahlen BD, Davis R, Dempster TA, Gerken H. Assessing the stability and techno-economic implications for wet storage of harvested microalgae to manage seasonal variability. Biotechnology for Biofuels. 2019 04-08;12(1):80.

3. Bessette AP, Teymouri A, Martin MJ, Stuart BJ, Resurreccion EP, Kumar S. Life Cycle Impacts and Techno-economic Implications of Flash Hydrolysis in Algae Processing. ACS Sustainable Chem Eng. 2018 Mar 5;6(3):3580–8.

4. Davis R, Wiatrowski M, Kinchin C, Humbird D. Conceptual Basis and Techno-Economic Modeling for Integrated Algal Biorefinery Conversion of Microalgae to Fuels and Products: 2019 NREL TEA Update: Highlighting Paths to Future Cost Goals via a New Pathway for Combined Algal Processing [Internet]. Golden, CO: National Renewable Energy Laboratory; 2020. Available from: https://www.nrel.gov/docs/fy20osti/75168.pdf

5. Talbot C, Garcia-Moscoso J, Drake H, Stuart BJ, Kumar S. Cultivation of microalgae using flash hydrolysis nutrient recycle. Algal Research. 2016 09-01;18:191–7.

6. DeRose K, DeMill C, Davis RW, Quinn JC. Integrated techno economic and life cycle assessment of the conversion of high productivity, low lipid algae to renewable fuels. Algal Research. 2019 03-01;38:101412.

7. Davis R, Kinchin C, Markham J, Tan E, Laurens L, Sexton D, et al. Process Design and Economics for the Conversion of Algal Biomass to Biofuels: Algal Biomass Fractionation to Lipid- and Carbohydrate-Derived Fuel Products [Internet]. National Renewable Energy Lab. (NREL), Golden, CO (United States); 2014 09-01. Available from: https://www.osti.gov/biblio/1159351

8. Davis RE, Grundl NJ, Tao L, Biddy MJ, Tan EC, Beckham GT, et al. Process Design and Economics for the Conversion of Lignocellulosic Biomass to Hydrocarbon Fuels and Coproducts: 2018 Biochemical Design Case Update; Biochemical Deconstruction and Conversion of Biomass to Fuels and Products via Integrated Biorefinery Pathways [Internet]. National Renewable Energy Lab. (NREL), Golden, CO (United States); 2018 11-19. Available from: https://www.osti.gov/biblio/1483234

9. Davis RW. Bioconversion of Algal Carbohydrates and Proteins to Fuels. DOE Bioenergy Technologies Office (BETO) 2019 Project Peer Review; 2019.

10. Monroe E, Shinde S, Carlson JS, Eckles TP, Liu F, Varman AM, et al. Superior performance biodiesel from biomass-derived fusel alcohols and low grade oils: Fatty acid fusel esters (FAFE). Fuel. 2020 05-15;268:117408.

11. Humbird D, Davis R, Tao L, Kinchin C, Hsu D, Aden A, et al. Process Design and Economics for Biochemical Conversion of Lignocellulosic Biomass to Ethanol: Dilute-Acid Pretreatment and Enzymatic Hydrolysis of Corn Stover [Internet]. National Renewable Energy Lab. (NREL), Golden, CO (United States); 2011 03-01. Available from: https://www.osti.gov/biblio/1013269-process-design-economics-biochemical-conversion-lignocellulosic-biomass-ethanol-dilute-acid-pretreatment-enzymatic-hydrolysis-corn-stover

12. Barrett JA, Jones ZR, Stickelmaier C, Schopp N, Ford PC. A Pinch of Salt Improves n-Butanol Selectivity in the Guerbet Condensation of Ethanol over Cu-Doped Mg/Al Oxides. ACS Sustainable Chem Eng. 2018 Nov 5;6(11):15119–26.

13. Gabriëls D, Yesid Hernández W, Sels B, Voort PVD, Verberckmoes A. Review of catalytic systems and thermodynamics for the Guerbet condensation reaction and challenges for biomass valorization. Catalysis Science & Technology. 2015;5(8):3876–902.

14. Liu F, Wu W, Tran-Gyamfi MB, Jaryenneh JD, Zhuang X, Davis RW. Bioconversion of distillers’ grains hydrolysates to advanced biofuels by an Escherichia coli co-culture. Microbial Cell Factories. 2017 Nov 9;16(1):192.

15. Hannon JR, Lynd LR, Andrade O, Benavides PT, Beckham GT, Biddy MJ, et al. Technoeconomic and life-cycle analysis of single-step catalytic conversion of wet ethanol into fungible fuel blendstocks. Vol. 117, Proceedings of the National Academy of Sciences. 2020. p. 12576–83.

16. Romero-Izquierdo AG, Gómez-Castro FI, Gutiérrez-Antonio C, Hernández S, Errico M. Intensification of the alcohol-to-jet process to produce renewable aviation fuel. Chemical Engineering and Processing - Process Intensification. 2021 Mar 1;160:108270.

17. Luning Prak DJ, Jones MH, Trulove P, McDaniel AM, Dickerson T, Cowart JS. Physical and Chemical Analysis of Alcohol-to-Jet (ATJ) Fuel and Development of Surrogate Fuel Mixtures. Energy Fuels. 2015 Jun 18;29(6):3760–9.

18. Geleynse S, Brandt K, Garcia‐Perez M, Wolcott M, Zhang X. The Alcohol-to-Jet Conversion Pathway for Drop-In Biofuels: Techno-Economic Evaluation. ChemSusChem. 2018;11(21):3728–41.

19. Tan ECD, Snowden‐Swan LJ, Talmadge M, Dutta A, Jones S, Ramasamy KK, et al. Comparative techno-economic analysis and process design for indirect liquefaction pathways to distillate-range fuels via biomass-derived oxygenated intermediates upgrading. Biofuels, Bioproducts and Biorefining. 2017;11(1):41–66.

20. Carlini C, Flego C, Marchionna M, Noviello M, Raspolli Galletti AM, Sbrana G, et al. Guerbet condensation of methanol with n-propanol to isobutyl alcohol over heterogeneous copper chromite/Mg–Al mixed oxides catalysts. Journal of Molecular Catalysis A: Chemical. 2004 Oct 11;220(2):215–20.

21. Perez CF, Orio OA, Herrero ER, Chiesa GR, Lavezzo MA. Dehydration of Amyl Alcohols (From Fusel Oil) Using Argentine Bentonites as Catalyst. Latin American Applied Research. 1988;18:139–44.

22. Hasan Z, Hwang J-S, Jhung SH. Liquid-phase dehydration of 1-phenylethanol to styrene over sulfonated D-glucose catalyst. Catalysis Communications. 2012 Sep 5;26:30–3.
